# P-1580. Evaluation of Shorter Versus Longer Antifungal Treatment Durations for Candida Urinary Tract Infections

**DOI:** 10.1093/ofid/ofae631.1747

**Published:** 2025-01-29

**Authors:** Wesley D Kufel, Jacob C Govel, Robert Seabury, Ramiro Gutierrez, Elizabeth Asiago-Reddy

**Affiliations:** Binghamton University School of Pharmacy Sciences, Binghamton, NY; Binghamton University School of Pharmacy and Pharmaceutical Sciences, Schenectady, New York; SUNY Upstate University Hospital, Syracuse, NY; SUNY Upstate Medical University, Syracuse, New York; SUNY Upstate Medical University, Syracuse, New York

## Abstract

**Background:**

Infectious Diseases Society of America guidelines recommend 14 days of antifungal therapy for Candida urinary tract infections (UTIs). For bacterial UTIs, studies have demonstrated that shorter antibiotic durations have comparable outcomes to longer durations. To our knowledge, there are no data to compare shorter (7 days) versus longer (14 days) antifungal durations for Candida UTI.

**Figure:**
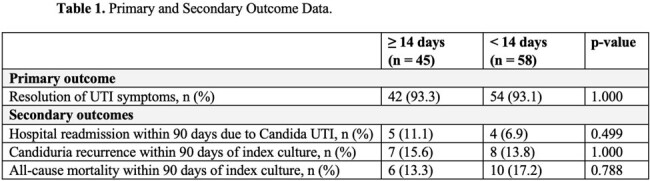

**Methods:**

This was a single-center, retrospective cohort study between 1/1/15 and 1/1/24 at a 752-bed academic medical center. Inclusion criteria were hospitalized adults with >1 urine culture with Candida spp. and symptoms (dysuria, increased frequency/urgency, hematuria, costovertebral angle tenderness, and/or fever) who received >1 antifungal dose within 96 hours of index culture (IC). Patients were excluded if Candida spp. were isolated from another culture site, received antifungals for a suspected non-UTI, or did not have any UTI symptoms. The primary outcome was to compare symptom resolution between shorter versus longer antifungal durations. Secondary outcomes included hospital readmission due to Candida UTI, candiduria recurrence, and all-cause mortality within 90 days of IC. Categorical and continuous variables were compared using Chi-square/Fisher exact test and the Mann-Whitney U test, respectively.

**Results:**

There were 2399 patient encounters with candiduria screened. After inclusion and exclusion criteria were applied, 103 patients were included for analysis with 45 and 58 in the >14 day and < 14-day cohorts, respectively. No significant differences were identified between cohorts in demographics, urologic procedures, urinary devices, nephrolithiasis, vasopressor use, mechanical ventilation, or hospital length of stay. ID consultation occurred in 60% and 62.1% of the >14 day and < 14-day cohorts, respectively, and all recommended antifungal treatment. Median (IQR) fluconazole duration was 14 days (14-14) and 7 days (5-7). No significant differences were identified in the primary and secondary outcomes (Table 1).

**Conclusion:**

No significant differences in treatment outcomes were identified between patients who received a median of 7 days versus 14 days of fluconazole for Candida UTI supporting shorter antifungal durations.

**Disclosures:**

**Wesley D. Kufel, Pharm.D., BCPS, BCIDP**, Merck & Co.: Grant/Research Support|Shionogi, Inc: Grant/Research Support **Elizabeth Asiago-Reddy, MD**, Abbvie: Grant/Research Support|GSK/Viiv: Grant/Research Support|Pfizer: Grant/Research Support|Theratechnologies, Inc.: Grant/Research Support

